# Ethical Principles in Patient-Centered Medical Care to Support Quality of Life in Amyotrophic Lateral Sclerosis

**DOI:** 10.3389/fneur.2019.00259

**Published:** 2019-03-22

**Authors:** Dorothée Lulé, Andrea Kübler, Albert C. Ludolph

**Affiliations:** ^1^Department of Neurology, University of Ulm, Ulm, Germany; ^2^Interventional Psychology, Psychology III, University of Würzburg, Würzburg, Germany

**Keywords:** ethics, quality of life (QoL), care, amyotrophic lateral sclerosis (ALS), well-being, depression, coping, psychosocial adaptation

## Abstract

It is one of the primary goals of medical care to secure good quality of life (QoL) while prolonging survival. This is a major challenge in severe medical conditions with a prognosis such as amyotrophic lateral sclerosis (ALS). Further, the definition of QoL and the question whether survival in this severe condition is compatible with a good QoL is a matter of subjective and culture-specific debate. Some people without neurodegenerative conditions believe that physical decline is incompatible with satisfactory QoL. Current data provide extensive evidence that psychosocial adaptation in ALS is possible, indicated by a satisfactory QoL. Thus, there is no fatalistic link of loss of QoL when physical health declines. There are intrinsic and extrinsic factors that have been shown to successfully facilitate and secure QoL in ALS which will be reviewed in the following article following the four ethical principles (1) Beneficence, (2) Non-maleficence, (3) Autonomy and (4) Justice, which are regarded as key elements of patient centered medical care according to Beauchamp and Childress. This is a JPND-funded work to summarize findings of the project NEEDSinALS (www.NEEDSinALS.com) which highlights subjective perspectives and preferences in medical decision making in ALS.

## Quality of life in ALS

Amyotrophic lateral sclerosis (ALS) is among the most devastating neurological conditions: patients lose the ability to speak, to walk and eventually to breathe. On average, patients die within 3 years after symptom onset. If life-sustaining measures such as invasive ventilation are taken, patients may terminate in a locked-in state with a clear mind in a paralyzed body. There is no cure for ALS and care focuses on maintaining functional ability and providing palliative and symptomatic interventions to relieve the burden of symptoms (1). The communication of the diagnosis is a major stressful event for patients, families and caretakers and thus most challenging with regard to medical counseling (2).

There are different ways of how patients cope with these major changes. Quality of life (QoL) is one possible measure of good psychosocial adaptation to disability such as ALS, similarly to depression (3). There have been contradictory reports whether QoL is lost in the course of physical decline (4–6). This discrepancy is partly attributed to selection of patient subgroups (e.g., shortly after diagnosis vs. long-term survivors) and the use of different QoL definitions. QoL is the general well-being of a person and includes physical (individuals' perception of their physical state), psychological (individuals' perception of their cognitive and affective state) and social dimensions (individuals' perception of the interpersonal relationships and social roles in their life). It is therefore not simply a state of physical integrity (7). QoL is increasingly used to supplement objective clinical or biological measures to evaluate health care provision and interventions in research and clinical trials (8).

There is a debate which QoL measure is truly patient centered. Measures are either based on hedonic concepts focusing on subjective factors and emotional evaluation or eudaimonic concepts with more objective factors of QoL such as physical health or economic status (9). As physical health declines in ALS and mobility becomes heavily restricted, these QoL measures provide evidence for low QoL in ALS simply by the nature of the underlying concept. These clinimetric endpoints are increasingly considered overly reductionistic (10) as they include aspects, which are no longer relevant or are out of range of an immobile patient, e.g., physical activity (11); thus, patients often prefer more subjective scales of QoL as these better capture their emotional state of well-being (Table 1). They might as well be regarded as possible outcome measures in clinical trials to determine the subjective benefit of a treatment for a patient. Observations concerning hedonic QoL are often counter-intuitive: simultaneous deterioration of physical integrity and well-being does not necessarily occur (19). Accordingly, ALS patients may experience a surprisingly high subjective QoL and an only moderately increased affective state as compared to healthy subjects (6, 20–27) which can be maintained throughout the course of ALS (27–29). This may even be true in the final state of complete immobility, the locked-in state [LIS; (30, 31)].

**Table 1:** Examples of most widely used measures of subjective, patient-centered QoL.

**Abbreviation**	**Measure**	**Procedure**	**Outcome**
**MEASURES OF GLOBALE SUBJECTIVE QoL**			
ACSA (12)	Anamnestic comparative self-assessment	Culturally independent and well-tolerated measure of general QoL; patient is asked to rate his or her current QoL on a scale from −5 to +5. Minus 5 indicates the worst, plus 5 the best ever experienced QoL. It is thus, a rating within each individual's own framework of QoL	ACSA score between −5 to +5
SEIQoL(-DW) (13)	Schedule for the Evaluation of Quality of Life direct weighting	Overall subjective QoL as judged by the patient through a semi-structured interview. The patients have to (1) name the life areas which are important to their QoL, (2) rate the current level of importance of each area and (3) rate the satisfaction with each of the areas	SEIQoL-Index-Score between 0 and 100%
Ganzini QoL (14)	QoL-single-item question	Single-item question to assess patients self-perceived overall QoL with end-points labeled 1 = “my quality of life is as good as it can be” and 6 = “my quality of life is very bad, horrible.”	Score between 1 and 6
Krampe QoL (15)	QoL-single-item question	Single-item question to assess patients self-perceived overall QoL with end-points labeled: “Over the past 7 days, the quality of my life has been”: very poor (0)–excellent (10).	Score between 0 and 10
ALSSQoL (16)	ALS-Specific Quality of Life Questionnaire	Fifty item disease-specific questionnaire on 6 domains adressing (1) Negative Emotion; (2) Interaction with People and the Environment; (3) Intimacy; (4) Religiosity; (5) Physical Symptoms; (6) Bulbar Function	Average total QOL score, and 6 domain scores
**MEASURES OF GLOBALE SUBJECTIVE QoL COMBINED WITH PHYSICAL QoL**			
WHOQOL-BREF (17)	Short version of the World Health Organization Quality of Life (WHOQOL)-Group questionnaire	Twenty-six item non-disease specific questionnaire on Physical, Psychological, Social Relations, Environment within cultural context	Domain scores between 0 and 100
MQoL (18)	The McGill Quality of Life Questionnaire	Subjective QoL according to five subscales: physical function, physical well-being, psychological symptoms, existential well-being and social support	MQoL score as mean of 5 subscales between 0 and 10
	Including MQOL single-item scale (SIS)	Single-item Score (SIS) of the MQoL for overall QoL on a visual analog scale	MQoL SIS score between 0 and 10

**Figure 1:**
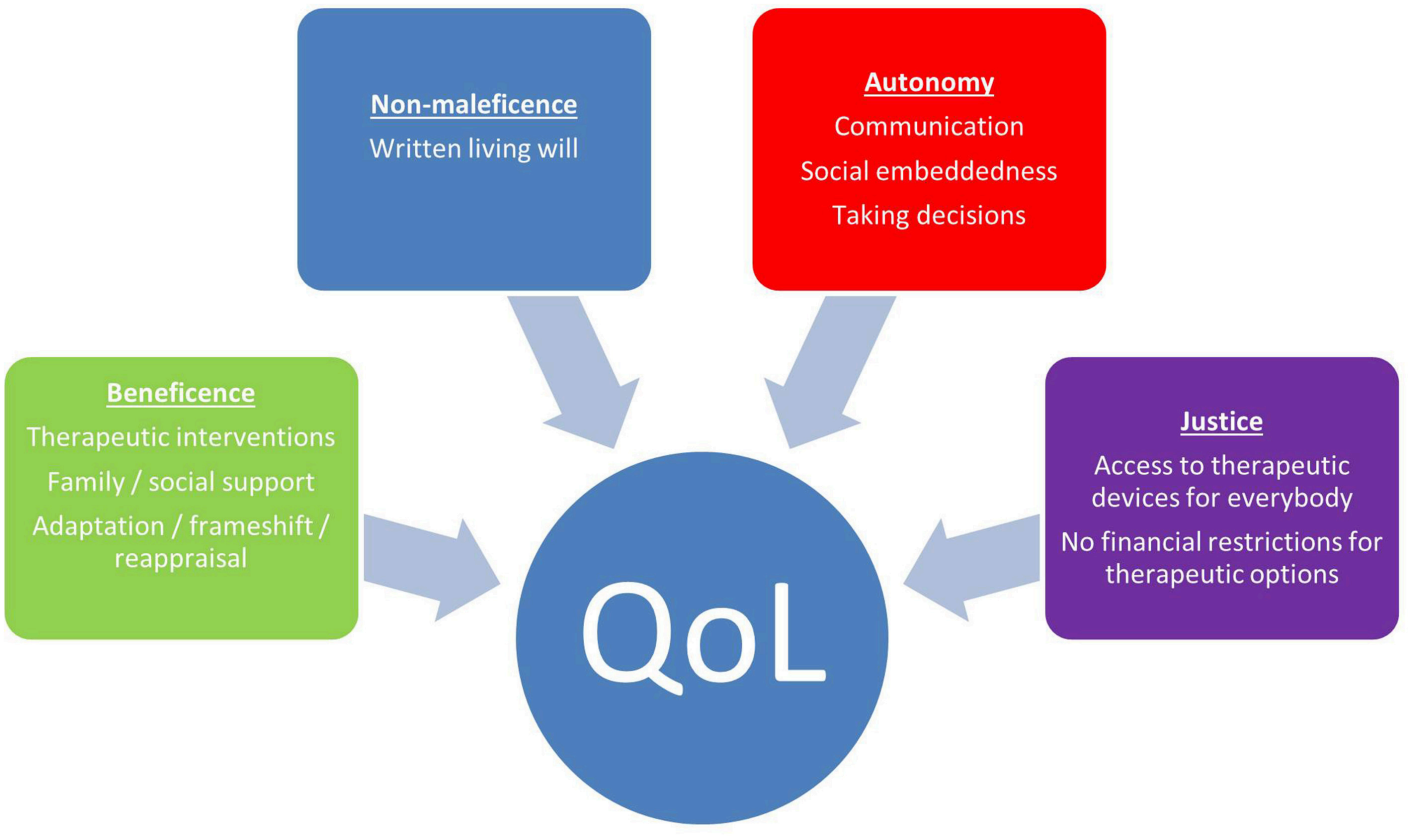
Factors according to medical ethics which facilitate QoL in ALS.

The lack of association of severity of illness and subjective QoL has been shown for several diseases and is referred to as the “*well-being-paradox*” (32). Prerequisite for this paradox is a process of psychosocial adaptation to the altered circumstances of severe physical function loss. According to the theory of homeostasis in quality of life, everybody has his/her individual level of well-being which he/she aims to reach which is usually in the range of 70–80% of the maximum QoL (33). Provided that sufficient time (29) and intrinsic (e.g., successful coping) and extrinsic resources (e.g., strong family support) are given, patients may show a process of ongoing change and adaptation of their expectations to the actual circumstances [TOTE model; (34)]. The capability of adaptation is not simply a matter of disease state or general personality traits (21). It can be successfully supported by medical teams through patient centered medical care. The different intrinsic and extrinsic factors in medical care to facilitate QoL in ALS and the individualistic perspective in medical decision making have been evaluated within the JPND-funded project NEEDSinALS (www.NEEDSinALS.com). These factors may be subsumed under the four ethical principles of good medical care according to Beauchamp and Childress (35), namely beneficence, non-maleficence, autonomy and justice (Figure 1).

### Beneficence

This principle requires that everything should be done in the best interest of the patient. Therapeutic interventions are usually introduced by the physician and their interdisciplinary teams to facilitate QoL in ALS. No cure is available yet, but different therapeutic interventions e.g., non-invasive ventilation (NIV) may be means also to prolong survival (36). Previous studies have provided extensive evidence that ALS patients with NIV have an increased QoL (20, 37, 38). Permanent respiratory insufficiency may lead to disturbed sleep, fatigue and reduced physical fitness, all these symptoms may be relieved by ventilation (39). Thus, ventilation may positively impact QoL and patients with ventilation may show even higher QoL than those without (20). Further, nutritional support is a major element of beneficence in ALS as a loss in BMI is a negative prognostic factor (40). Unfortunately, fear of choking during meals is widely prevalent in patients with bulbar symptoms (41), so many patients fear to eat at all. Thus, introduction of a PEG may be a highly useful approach to improve QoL as it allows for weight control while relieving the patient from the pressure to eat. As patients may nevertheless be able to have oral food intake, the pleasure and sensuality of eating can be maintained which additionally supports QoL. However, in some patients the positive effect of PEG insertion might be outbalanced by “particularly strong feelings of loss of control” (41), highlighting the subjective perspective on patient centered care (8). Other therapeutic interventions may as well-facilitate QoL such as application of botox to stop the debilitating syndrome of “drooling” (sialorrhoea). Also, therapies such as physiotherapy, occupational therapy (ergotherapy) and speech therapy (logopedics) may relieve physical symptoms of pain, muscle tension and stiffness which all finally may help to improve QoL (42).

Apart from therapeutics, there is one major extrinsic factor which may substantially improve QoL which is social support (23). Family is the most frequently named aspect of individual QoL in ALS (26, 29, 43). And, as satisfaction with family was often good among patients, the patient's QoL is also often good (44). For healthy subjects, there are other factors which are important for well-being (finances, career) whereas most ALS show a response shift toward social support in the course (20). Thus, inclusion of family members in clinical counseling and supporting the patient in refocusing on social resources may facilitate QoL.

Apart from these extrinsic factors, QoL in serious illness is highly depending on intrinsic factors, such as resilience which is a general characteristics addressing the capacity to recover quickly from difficulties (45). In this context, there has been evidence in ALS that appraisal of coping potential and mental attitudes may be crucial to adapt (23). A re-set of preferences referred to as response shift (46) may support these inner processes with the ability to see what is still there and what is untouched by the disease [e.g., spiritual well-being; (20)]. Further, reframing, the ability to see the same situation from a different perspective [e.g., instead of looking at what you lose, you pay attention to what is spared such as your emotions, feelings and desires; (20)] can be highly supportive for psychosocial adaptation. Finally, many years of research about adapting to and living with chronic diseases suggest that mindfulness in the sense of accepting the circumstances which cannot be changed without judgement and focus on the present (47) may reduce the negative psychological impact of the illness (48, 49).

Patients can be encouraged to use these inner resources mentioned above. Psychotherapeutic interventions may help to improve the QoL of patients and may even prolong survival as the psychoemotional state of the patient has impact on QoL (50) and survival time (49). The beneficence of the above mentioned intrinsic factors may as well be addressed in clinical routine by physicians and medical staff to encourage patients to give more room in life for any beneficial intrinsic process.

Beneficence requires the knowledge of the patient's wishes as peer evaluations might not meet the patient's actual needs. There is evidence for discrepancy between patient's well-being and the perspective on patient's well-being of people without neurodegenerative conditions (27, 29). Peers judgement of patient's well-being is primarily based on personal opinion when they anticipate a low QoL in severely disabled patients. This is true for people without experience in ALS and is even true for caregivers and physicians if they lack experience with ALS (51). Healthy subjects may be blind toward the patient's process of ongoing change and adaptation and instead they may conclude from their personal perspective. The more experienced healthcare professionals are, the more they know about the capacity to adjust and are thus abled to correctly anticipate patient's quality of life, affective state, and wish for hastened death (51).

Thus, the knowledge of and believe in beneficence in ALS is a matter of the physician's experience. As many patients gradually adjust to their situation and also possibly change their therapeutic preferences in the course of the disease (52), beneficence from the patient's perspective is a dynamic construct which needs to be recognized and may be supported by caretakers and medical care teams.

### Non-maleficence

*Primum non-nocere*, refrain from harm is the other side of the coin of beneficence and thus, similar aspects concern maleficence than beneficence. Non-maleficence needs to be considered the moment the diagnosis is communicated. “Breaking the news” is a highly delicate balance between patient's need to be informed which requires veracity and fidelity on the one hand and the right for denial on the other hand which can be a helpful strategy at least shortly after diagnosis (23). Maleficence in the sense of the emotional burden of diagnosis can be reduced by using a thorough approach for breaking the news as it may attenuate negative impact on QoL (53). But also providing sufficient information can prevent maleficence: patients with sudden respiratory insufficiency in an emergency situation who have been informed on all aspects of respiratory support may feel more competent to take the right decision (41).

Advance directives and living wills are crucial to prevent maleficence, e.g., insertion of a tracheostomy in an emergency if the patient does not want to [possibly because he/she is afraid of the burden for others; (52)]. Many therapeutic options secure QoL (e.g., ventilation) but most patients are unable to anticipate this shortly after diagnosis. During the course of ALS, some might dismiss the idea of maleficence of invasive ventilation and might realize the beneficial effect of this therapeutic treatment (29). Therefore, dynamic adjustment to living wills is a key aspect to prevent maleficence.

Preferences regarding therapeutic measures are highly determined by patient's personal values, religious beliefs and cultural background (54).

Cultural differences exist: in Japan, invasive ventilation is more regarded as routine therapeutic treatment than in other countries [33% in Japan; (55)]. In some countries, life prolongation might be regarded as maleficence and more life-shortening treatments are suggested (56). Thus, social context may define what is beneficence and maleficence in the context of cultural norms (54, 57).

### Patient's Autonomy

Patient's sense of autonomy is a key issue of quality of life and goes beyond being physically autonomous to perform an action. Autonomy also encompasses the sense of capability to take decisions and the feeling of being an author of one's own action which is a key feature of self-efficacy and thus for QoL (58). Taking decisions also sustains the feeling of social embeddedness disregarding physical disability, e.g., the patient can be included in family decisions and may participate in daily routine if possible (59). This allows the patient to be an active part of daily routine: to participate in decision making, to be asked questions, to express concerns, address fears and anxieties, express wishes, values, desires, and hopes. It is noteworthy that possible minor cognitive deficits in some patients do not interfere with the competency to decide and participate (60).

It is especially challenging to secure patient's autonomy in LIS as there is lack of direct means to communicate in this state. Assisted communication (20, 29) becomes important for individual QoL in the course of ALS, but is not mentioned by patients in early stages of the disease (20). Many patients use letter board for communication which requires considerable effort from a second person to record which item the patient selected from the board. Technical devices may allow for communication but these are time consuming and strenuous to use and also additional assistance is required (59). Thus, knowledge on patient's wishes, desires and thoughts in advanced stages is sparse and there is substantial lack of understanding which factors may impact the dynamics of QoL and affective state in the course of ALS (30). Communication via eye-gaze control is possible, including standardized interviews (61, 62) but the latter are rarely performed. Brain Computer Interfaces (BCI) are promising technologies for communication and interaction (63, 64) but in a subgroup of patients only (65). Other means of BCI-use such as unrestricted access to web browsers of which some are adaptable to home based BCI systems (66) secure new degrees of freedom in severe paralysis (67–70). Some patients already use these techniques in their home environment for communication and painting (71–76) and first evidence support the notion that these techniques positively impact QoL (71, 77). In the future, with major advances in communication technology well-being in ALS might possibly be facilitated. BCIs might also be indirectly used in evaluation and recognition of well-being and emotional state in highly advanced patients (78) such as the amplitude of the N400, a negative deflection of the EEG curve following a meaningful event (79), which was higher in patients with high QoL compared to those with a poor. Thus, the N400 may serve as an objective physiological indicator of individual QoL in non-responsive ALS patients (80). Overall, there is still a long way to go until BCI will be a standard tool for home care for a majority of ALS patients (63, 81). But for patient-centered care, compensation for progressive loss of verbal speech is mandatory to secure patient's autonomy and QoL (52).

### Justice

This ethical principle of care requires that all patients are treated in an equal way without prejudice or social discrimination. In the sense of justice, patients in similar situations should have access to the same care options. Palliative care intervention improves quality of life in patients and caregivers (82) and medical care may facilitate this positive dynamics by offering this care to every patient. ALS patients expect dignified care (82) but instead, patients are often dissatisfied with health care services (83). Every patient needs to be treated differently according to the actual preferences and needs (54). There is no justice in defining every person by the diagnosis with a nihilistic view of the disease which has to be prevented under all circumstances (48). Instead, to grant justice every patient has to be regarded as an individual with specific needs and the right to be treated the same according to his/her preferences, disregarding mental, societal or financial status.

Further, providing sufficient information according to the patient's needs as outlined above is also a matter of justice. Thus, granting the patient the right for information is similarly a matter of justice as granting the right for not-knowing. In this sense, it is a matter of justice to grant patient's will even if it interferes with the physician's personal and professional opinion.

**Figure 2:**
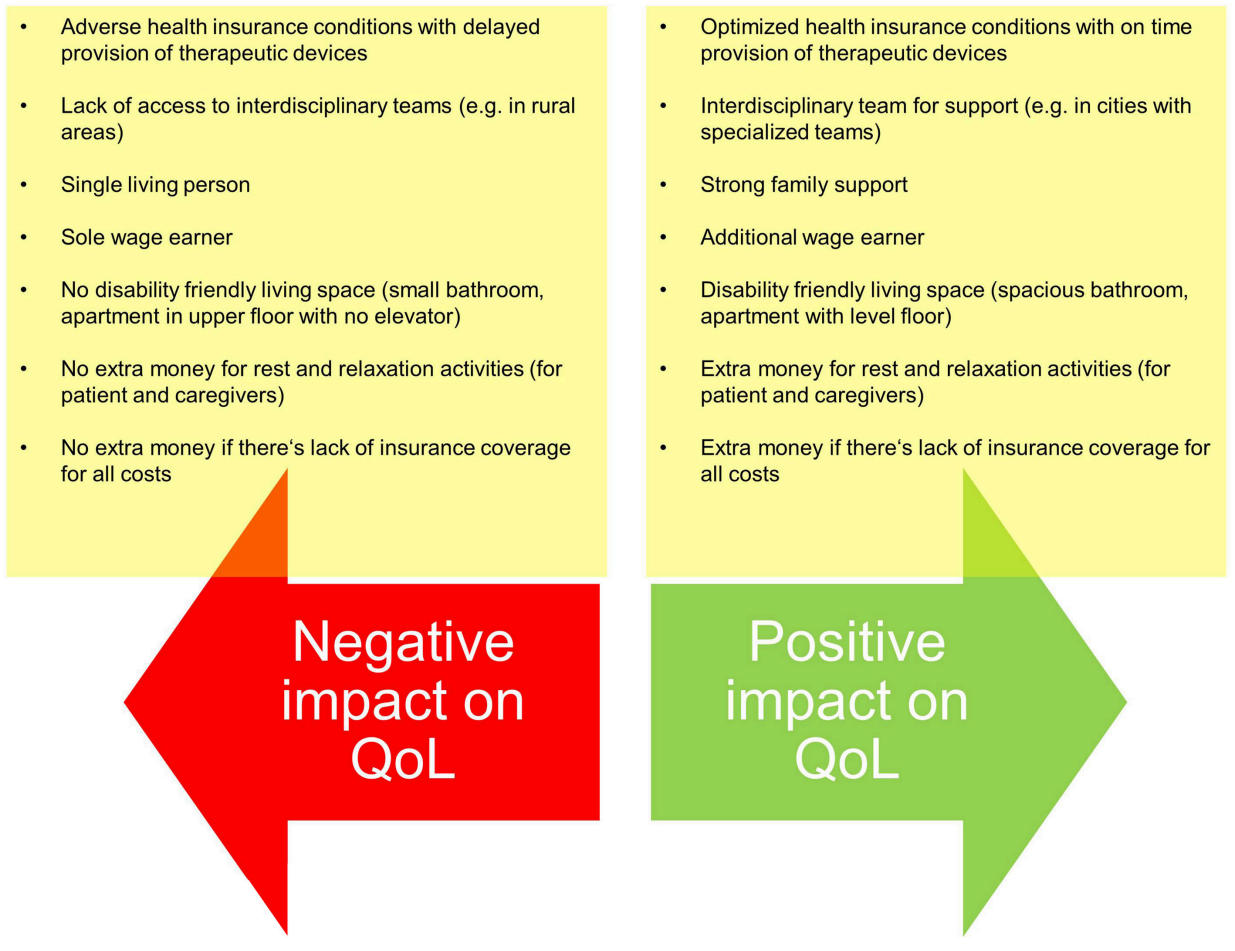
Examples of setting differences which may interfere with justice in medical care despite optimized care provision by the medical team.

Finally, justice in medical care is secured in many countries as most healthcare systems secure this kind of justice by providing coverage of (most) costs. Despite that most medical systems are based on a solidary idea allowing for justice, the impact of the disease may vary between patients thus justice in clinical care is not easy to accomplish. There are basic settings which significantly hamper justice in care provision which cannot be changed by the medical team, e.g., there is variance of the paid costs by the insurance companies. Further, in some instances, only basic technical equipment is provided which possibly don't meet the patient's actual needs. Thus, patients have to cover the extra charges for the devices which fully meet their needs. And finally, there are personal settings (e.g., living and working conditions) which may heavily impact patient's life with ALS and which interfere with the principle of justice (Figure 2).

## Consequences and Future Directions

There is evidence that considerate medical care within multidisciplinary teams (84) helps patients to find their own way of coping with the disease to gain or maintain a satisfactory QoL (48). Living with a fatal disease creates a crisis loaded environment and adapting to the disease is a psychological process rendering mandatory a strong support from these specialists' teams (41). Following the ethical principles of medical care as outlined in this text allows for a holistic support of the patient to secure QoL.

## Data Availability

The raw data supporting the conclusions of this manuscript will be made available by the authors, without undue reservation, to any qualified researcher.

## Author Contributions

DL, AK, and AL have collected data underlying this review and discussion of data. Text writing was done by DL, thorough revision of the manuscript was performed by all authors.

### Conflict of Interest Statement

The authors declare that the research was conducted in the absence of any commercial or financial relationships that could be construed as a potential conflict of interest.
